# Directed evolution of a filamentous fungus for thermotolerance

**DOI:** 10.1186/1472-6750-9-74

**Published:** 2009-08-26

**Authors:** Eudes de Crecy, Stefan Jaronski, Benjamin Lyons, Thomas J Lyons, Nemat O Keyhani

**Affiliations:** 1 Evolugate LLC, 2153 SE Hawthorne Road, 15 Gainesville, FL, 32641, USA; 2 USDA ARS NPARL, 1500 N. Central Ave., Sidney MT 59270, USA; 3 Department of Microbiology and Cell Science, University of Florida, Gainesville, FL 32611, USA

## Abstract

**Background:**

Filamentous fungi are the most widely used eukaryotic biocatalysts in industrial and chemical applications. Consequently, there is tremendous interest in methodology that can use the power of genetics to develop strains with improved performance. For example, *Metarhizium anisopliae *is a broad host range entomopathogenic fungus currently under intensive investigation as a biologically based alternative to chemical pesticides. However, it use is limited by the relatively low tolerance of this species to abiotic stresses such as heat, with most strains displaying little to no growth between 35–37°C. In this study, we used a newly developed automated continuous culture method called the Evolugator™, which takes advantage of a natural selection-adaptation strategy, to select for thermotolerant variants of *M. anisopliae *strain 2575 displaying robust growth at 37°C.

**Results:**

Over a 4 month time course, 22 cycles of growth and dilution were used to select 2 thermotolerant variants of *M. anisopliae*. Both variants displayed robust growth at 36.5°C, whereas only one was able to grow at 37°C. Insect bioassays using *Melanoplus sanguinipes *(grasshoppers) were also performed to determine if thermotolerant variants of *M. anisopliae *retained entomopathogenicity. Assays confirmed that thermotolerant variants were, indeed, entomopathogenic, albeit with complex alterations in virulence parameters such as lethal dose responses (LD_50_) and median survival times (ST_50_).

**Conclusion:**

We report the experimental evolution of a filamentous fungus via the novel application of a powerful new continuous culture device. This is the first example of using continuous culture to select for complex phenotypes such as thermotolerance. Temperature adapted variants of the insect-pathogenic, filamentous fungus *M. anisopliae *were isolated and demonstrated to show vigorous growth at a temperature that is inhibitory for the parent strain. Insect virulence assays confirmed that pathogenicity can be retained during the selection process. In principle, this technology can be used to adapt filamentous fungi to virtually any environmental condition including abiotic stress and growth substrate utilization.

## Background

Filamentous fungi are among the most widely used whole cell biocatalysts in a host of agricultural, food, environmental and bioenergy related applications [1,2]. Not surprisingly, there is tremendous interest in manipulating the genetics of fungi to improve their industrial performance. Unfortunately, traditional methods of genetic engineering are expensive, slow and manually intensive. Moreover, fungi possess complex regulatory circuits that intimately control cellular growth and metabolism. Consequently, single, or even multiple directed mutations often fail to produce the desired results. Newer methods, such as gene prospecting [3], have been developed that are higher throughput, however such methods are still limited by the fact that many favorable phenotypes, like sustained thermotolerance, often require genome-wide changes [4] that are difficult to select for by inserting individual genes or even groups of genes. The only practical way to alter such complex phenotypes is using continuous culture to select for genetic variants that can adapt to gradual changes in environmental conditions – in essence, harnessing the power of evolution by natural selection [5].

There are two widely used methods for continuous culture, serial dilution and chemostats, both of which are plagued by serious limitations that prevent their use for experimental evolution. For a detailed discussion of these limitations see de Crécy et al., 2007 [6]. Serial dilution requires the periodic transfer of a random sampling of cells to a new vessel and, consequently, is troubled by contamination problems. While serial dilution has been used to experimentally evolve filamentous microbes on plates [7], this methodology cannot be used in liquid cultures because the cells form aggregates and individual cells cannot be randomly chosen for dilution. Chemostats and similar continuous culture devices are closed systems and are not plagued by contamination. Indeed, filamentous fungi have been cultured in chemostats [8]. However, filamentous fungi prefer to grow on solid surfaces and they readily adhere to the walls of chemostats, thus evading dilution as well as forming heterogeneous populations. Wall growth, even small amounts of it, limits the length of time cells can spend adapting in a chemostat, making the technology inadequate for long-term adaptation.

A new method of continuous culture, called the Evolugator™, has been developed that circumvents many of the problems associated with serial dilution and chemostat technology [6]. More importantly, the Evolugator™ is, in principle, capable of culturing filamentous fungi continuously *ad infinitum *without wall growth becoming problematic. This technology represents a critical breakthrough for industrial mycology, allowing the development of fungal strains for virtually any application.

Herein, we report the first application of the Evolugator™ technology for the evolutionary adaptation of filamentous fungi of commercial importance. The use of the entomopathogenic fungus *Metarhizium anisopliae *as a biological alternative to chemical pesticides has gained prominence and commercialized products based upon the fungus are available worldwide [9-11]. As a species, *M. anisopliae *has a broad host range that includes over 900 insects species and includes ticks, mites, and other members of the class *Arachnida*, although many individual strains have a narrower host range [9,12,13]. Commercial *M. anisopliae *formulations have been developed as biological control agents for various beetles, cockroaches, weevils, termites, flies, gnats, thrips and ticks [10,14]. In other countries, *M. anisopliae *has been applied for the control of grasshoppers, locusts, cockchafers, grubs, borers and even malaria-vectoring mosquitoes [15-19].

Despite their potential, however, several factors have hindered widespread adoption of fungi as part of biological control regimes. In particular, efficacy against certain insects is impeded by the relatively low resistance of these fungi to elevated temperatures [20,21]. Thermotolerance is important because some insects can elevate their body temperatures above ambient, particularly when diseased, either as part of their innate immune response or by basking in sunlight; a phenomenon termed "behavioral fever" [22-25]. Since the upper thermal limit for conidial germination and growth of many commercially useful fungi is generally 32–34°C [26-28], this represents an effective means of staving off fungal infections. It is also important to note that non-thermotolerant fungi are not effective in tropical and subtropical climates where ambient temperatures exceed those that permit fungal growth.

Not surprisingly, significant resources have been dedicated to developing methods for genetic engineering and other manipulation of *M. anisopliae *to improve its efficacy as a biocontrol agent [29-33]. Of particular interest is the development of thermotolerant strains that can both evade the host thermal response and retain efficacy in hot climates. This is the ideal problem to test the power of the Evolugator™ technology, which uses flexible tubing as a culture chamber. Since both the tubing and medium is changed with every dilution cycle, the Evolugator™ can, in principle, allow the growth and selection of fast-growing variants of filamentous fungal cells that could use the tubing as a solid surface upon which to grow [6]. As a proof-of-principle regarding the Evolugator™ technology, we adapted *Metarhizium anisopliae *strain ARSEF2575 (USDA ARS Insect Pathogenic Fungus Collection, Ithaca, NY), whose normal upper thermal limit for growth is 32°C, to grow at 37°C.

## Results

### Selection of thermostable *M. anisopliae *isolates

**Figure 1:**
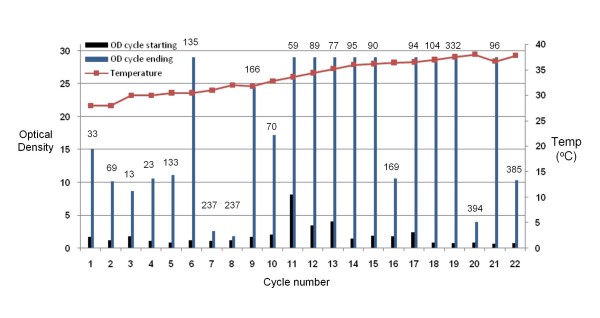
**Directed evolution of thermotolerant *M. anisopliae *isolates**. The temperature (red line), the starting OD (blue bar graph) and the OD at the time of initiation of the dilution cycle (green graph) are plotted versus the cycle number. The length of time (hr) of each cycle before dilution is shown above the OD values for each cycle.

An actively growing culture of *M. anisopliae *was inoculated inside the Evolugator™ growth chamber at 28°C as described in the Methods section. Growth was monitored by optical density (OD) and dilution cycles were initiated according to OD or cycle duration. Figure 1 presents a detailed description of 22 successive selection cycles over a 4 month period. For each cycle, the temperature of the culture chamber was recorded as well as the starting OD and ending OD. The starting OD is always low because the cells have just been diluted with fresh medium. The ending OD is higher because the cells have multiplied. Figure 1 also shows the duration of each dilution cycle, which is the length of time the cells are allowed to grow prior to initiating a new dilution cycle.

The fungus displayed rapid growth characteristics in cycles 1–4 where the temperature increased from 28°C to 30°C. During these cycles the culture duration was 1–2 days. Beginning at cycle 5, however, the growth rate slowed down – as evidenced by an increase in the amount of time it takes to grow enough cells to initiate a dilution. This indicated that it was taking longer for favorable variants to take over the population. Moreover, the maximal cell yield (OD) dropped significantly during cycles 7 (31°C) and 8 (32°C), even though cells were allowed to grow for over 200 hours each time, indicating decreased overall fitness. In cycles 8 and 9, the chamber temperature was not varied significantly in order to allow variants that can grow rapidly at this temperature to take over the population. Similar phenomena, where cycle duration needed to be increased and temperature stabilized to allow fast growing variants to take over, were also seen in cycles 16 (34.6°C) and 20 (38°C). Two strains, termed EVG016 and EVG017 were isolated from cells cultured in cycles 18 and 22, respectively. Sequencing of the ITS1 and a fragment of the *M. anisopliae *specific protease Pr1 genes revealed that both isolates were derivatives of the original wild type strain.

### Phenotypic characterization of *M. anisopliae *thermostable isolates

Isolates EVG016 and EVG017 were streaked on Potato-dextrose agar (PDA) plates. Wild-type *M. anisopliae *(2575) typically produces green pigmented spores (conidia) within 3–5 days of cultivation on these plates. EVG016 produced colonies that appeared less green than the wild-type, whereas EVG017 produced white colonies with occasional spores visible at colony fringes or at the center of the colony. Microscopic examination revealed reduced spore production in EVG017. Conidial production in replicated solid substrate fermentation confirmed reduced sporulation. EVG016 produced a mean of 7.7 × 10^11 ^conidia/kg barley substrate versus 3.9 × 10^12 ^for the parent strain, a statistically significant difference (P < 0.05, Student t-test). EVG017 produced less than 1% of the spores of the wild-type strain. We isolated a variant of EVG017, named EVG017g, that retained thermotolerance but was as capable of conidiation as wild type (See below).

**Figure 2:**
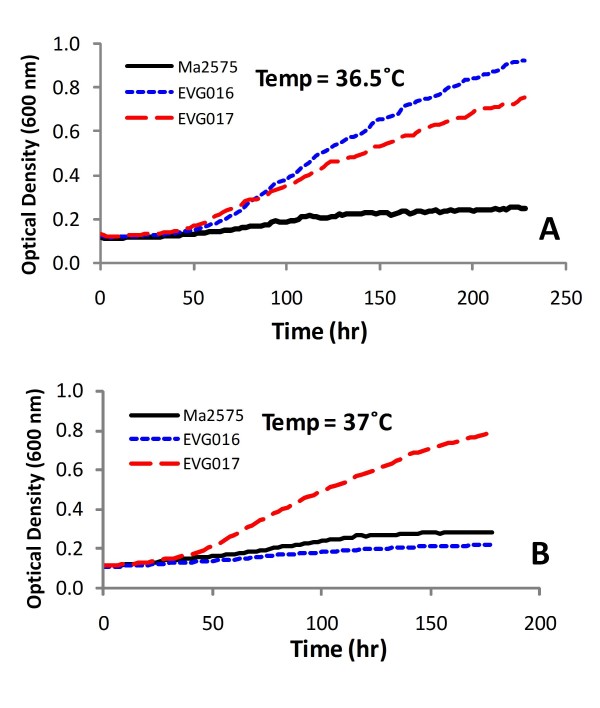
**Growth curves at 36.5°C (A) and 37°C (B) of wild-type and temperature adapted *M. anisopliae *isolates**. Growth experiments were performed using a high-throughput Bioscreen C plate reader and are the averages of at least 30 independent growth curves each for three independent batches of fungal conidia (>90 cultures).

**Figure 3:**
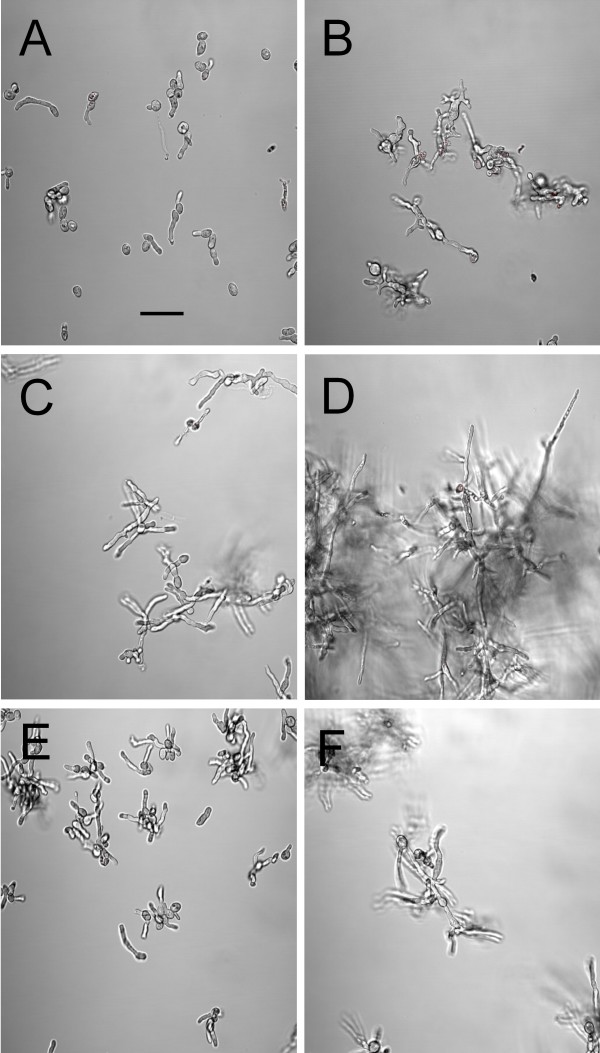
**Differential interference contrast (DIC) images of wild-type and temperature adapted *M. anisopliae *strains grown at 37°C**. EVG016 2 d (A) and 5 d (B), EVG017 2 d (C) and 5 d (D), Wild-type 2575 2 d (E) and 5 d (F). Bar = 20 μm.

The growth characteristics of the wild-type parent, EVG016 and EVG017 in liquid media were examined at various temperatures. All three strains displayed similar growth kinetics at 28°C, whereas only EVG016 and EVG017 displayed robust growth at 36.5°C (Figure 2). Only EVG017 grew at 37°C and no growth was evident for any of the strains at 38°C, indicating a narrow threshold for the adaptive response. In contrast, neither the wild-type nor the heat adapted strains displayed appreciable radial growth at 36–37°C when plated on solid (agar) media, although all displayed similar growth kinetics at 28°C. The strains did remain viable, and radial growth on plates was evident after a short lag period when plates were shifted from 37°C to 28°C **(data not shown)**. Microscopic examination of the growth of the adapted and wild-type strains revealed that whereas both the wild-type and EVG016 germinated and grew across the surface of the agar, EVG017 displayed more rapid formation of appressoria than the parent and the fungal hyphae of this strain appeared to begin to penetrate the agar during the initial stages of growth. The two adapted strains also displayed different hyphal morphologies. Microscopic examination of the growing cells (in liquid culture) revealed short-tubular growth of EVG016 at 37°C, whereas EVG017 at 37°C appeared similar to wild-type grown at 28°C (Figure 3). Interestingly, our results indicate that the wild-type strain was able to germinate at 37°C, but failed to subsequently grow.

### Insect bioassays

Insect bioassays against the Migratory Grasshopper, *Melanoplus sanguinipes*, were performed using the wild-type and adapted strains. Due to the reduced sporulation of EVG017, not enough spores could be directly harvested for insect bioassays. Therefore, the strain was passaged once through *M. sanguinipes *by rubbing the abdomen of host insects on an agar culture of EVG017. The fungus was then re-isolated from an insect cadaver after 6 d incubation and single spores isolated. The resultant strain, EVG017g, yielded satisfactory sporulation on solid substrate at 28°C (1.61 × 10^12 ^conidia/Kg barley), displayed the same growth kinetics and morphology as EVG017 (at 28°C and 37°C) and was therefore used for the insect bioassays.

**Figure 4:**
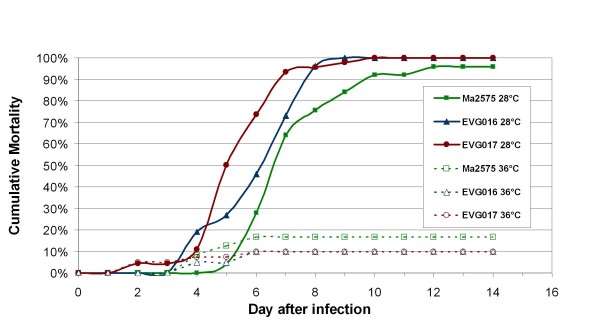
**Mortality assays of wild-type and temperature adapted *M. anisopliae *isolates**. Insect bioassays were performed using the migratory grasshopper, *Melanoplus sanguinipes *as described in the Methods section. The cumulative mortality over time is plotted for each strain with bioassays performed at 28°C and 36°C as indicated.

Infectivity and virulence of the wild type, EVG016 and EVG017g was evaluated using a topical 5-dose bioassay with doses bracketing the approximate LD_50 _based on exploratory assays (see Methods). Both EVG016 and EVG017g displayed lowered infectivity as expressed by greater LD_50 _values compared to the wild-type parent, although due to the slopes of the dose-response curves the effect was dramatically reduced at LD_95 _values (Figure 4, Table 1). Given that most biological control regimes require >90% mortality towards target insect in order to be effective, the latter values, may in fact be more important.

**Table 1:** Lethal dose response data derived from topical bioassays of the parent *M. anisopliae *ARSEF2575, EVG016 and EVG017g strains with adult *M. sanguinipes *grasshoppers at 28°C.

*M.a*. strain	Assay	LD_50_	95% CL	Slope (SE)	Chi Sqr	LD_95_	95% CL
2575	1	799	63–1,722	1.46 (0.51)	0.04	10,599	4,415–36,196
	2	1,815	1,174–3,042	1.60 (0.24)	1.55	19,503	9.279–68,978
	mean (S.D.)	1,307 (718)				15,051 (6,296)	
EVG016	1	25,453	19,600–41,000	3.73 (0.82)	0.75	70,257	50,534–138,856
	2	19,758	14,000–2.7400	2.55 (0.37)	2.89	87,347	57,234–169,968
	mean (S.D.)	22,605 (4,027)				78,802 (12,084)	
EVG017g	1	8,939	4,425–13,194	2.50 (0.64)	0.71	40,787	25,601–127,114
	2	14,007	4,365–25,838	1.62 (0.41)	2.35	145,180	71,373–765,860
	mean (S.D.)	11,473 (3,584)				92,983 (73,817)	

Units for LD and confidence levels: conidia/insect. Data are derived from two replicate bioassays using a total of 120–150 insects/bioassay.

Virulence at 28°C, in terms of Median Survival Time (ST_50_) calculated using Kaplan Meier Survivorship Analysis [34], showed overall significant differences among the three fungal strains (Logrank Test Chi Square 16.45, 2 df, p = 0.0003). EVG017g had a significantly faster kill (ST_50_), 5.5 d (95% Confidence Limits of 5.0–6.0 d), compared to 7 d (95% Confidence Limits of 7.0-7.0 d) for the wild-type parent (Logrank Test, S = -15.12; p = 0.0001), for a decrease of 20%. The ST_50 _value for EVG016, 6.0 d (95% Confidence Limits 6.0–7.0 d), was also significantly lower than that of the wild type (Logrank Test S = -9.0632, p = .025). EVG016 and EVG017g were not significantly different from each other (Logrank Test, S = 7.032; p = 0.063). The LD_50 _and ST_50 _of EVG017g may have been affected by its passage through and reisolation from a grasshopper. Nevertheless, EVG017g still demonstrated reduced infectivity as did EVG016. None of the strains were pathogenic or able to cause mortality in hosts at 36°C. However, when insects infected at 36°C were subsequently placed at 28°C, the hosts were rapidly killed by all three fungal strains, indicating that the wild type and adapted strains remained viable at 36°C, but could not cause pathogenicity and death.

## Discussion

Although there are a few technologies that can be used to experimentally adapt and/or select organisms for specific characteristics, these have been for the most part limited to short-term experiments. Both serial dilution and chemostats have critical limitations that make long-term evolutionary adaptation impossible. For serial dilution, this limitation is the high probability of contamination. For chemostats, this limitation is the "wall growth" problem. These problems are particularly problematic for continuously culturing adherent cells like filamentous fungi, although there are examples of both being used for the short term selection of filamentous fungi [7,8]. The Evolugator™ circumvents the contamination problem because the culture chamber is a continuous length of flexible tubing and cultures are never exposed to the outside. Furthermore, wall growth is not an issue for the Evolugator™ because the majority of the "wall" is replaced with each dilution. This means that cells can be cultured continuously for very long periods of time, allowing for the selection of complicated traits that cannot be achieved with short-term experimental evolution.

The Evolugator™ is the ideal method for selecting variants of filamentous fungi with complex phenotypes. The availability of a robust selection method for filamentous fungi is an important new tool for directed adaptation that could have significant applications ranging from improved industrial strains to the examinations of the mechanisms that underlie evolution in eukaryotic organisms. In this report, we demonstrate the successful adaptation of a filamentous fungus for thermotolerance as proof-of-principle that the technology can be applied for this purpose. Samples taken during the adaptation phase yielded two thermostable isolates with different phenotypic characteristics, although both were able to grow at 36.5°C, whereas the parental isolate could not. To our knowledge, this is the first report of the directed adaptation of a filamentous fungus for a complex phenotype via continuous culture.

Analysis of secondary non-selected traits, such as conidiation and virulence, revealed complex consequences of thermal adaptation. For example, EVG016 showed decreased infectivity when compared to wild type as measured by LD_50_, yet was not significantly less infective than wild type as measured by LD_95_. These results could simply be due to the long term culture of EVG016 in rich liquid media, conditions that are known to be able to cause attenuation of pathogenicity. However, the ST_50 _value for EVG016 was significantly lower than that of wild-type, *i.e*. it was a better pathogen. Absent additional thermotolerant isolates it is difficult to determine if the increased pathogenicity is associated with the thermotolerant phenotype or was a trait that was selected for serendipitously. EVG017, our second isolate from the same lineage, showed greatly impaired conidiation that could, in part, be offset or recovered by passage of the adapted isolate through an insect host. Remarkably, the resulting variant, EVG017g, maintained thermotolerance after passage through the insect and showed increased virulence compared to the non-insect passaged parent strain as measured by ST_50_. The LD_50 _remained higher than wild-type, but was lower than that of EVG016. The most likely explanation for these results is that the increased virulence of EVG017g was acquired during passage through the insect rather than during the thermal adaptation. Another possibility is that the increased infectivity (ST_50_) is an independent trait that arose in the lineage prior to the isolation of EVG016. A final possibility is that the enhanced infectivity is linked to the thermolerant trait. Much more work needs to be done to distinguish between these possibilities. However, we have shown that virulence can be recovered following loss due to the thermal adaptation protocol.

While the purpose of this experiment was to produce thermotolerant strains, the eventual goal is to produce better entomopathogens by generating variants that can sustain the insect thermal response. It is intriguing to speculate that the changes we measured in virulence parameters are related to the acquisition of thermotolerance. To test this, we reared the infected *M. sanguinipes *at 36–37°C to mimic the insects' ability to thermoregulate to a temperature that is the new upper threshold of the evolved strains. Measurements of body temperature revealed that the insects maintained a constant body temperature that was in equilibrium with the cage temperature (36–36.5°C). Despite their confirmed thermotolerance, the adapted variants did not show increased virulence at 36–37°C, indicating that the ability to grow *in vitro *at 36–37°C does not necessarily mean that *in vivo *growth and pathogenesis will occur.

It should be noted that the selection was not directed towards an insect target, simply for growth in a rich broth liquid medium at previously non-permissive temperatures. We hypothesize that insect target specificity and/or virulence might be increased via continuous culture adaptation on specific insect cuticular extracts as the growth substrate. Alternately, the technology can be applied to adapt strains for greater resistance to abiotic stresses such as UV-irradiation. All of these approaches can be employed be obtain better biocontrol agents via continuous culture.

From a population biology and genetic framework, within our experimental set-up any novel mutation in a population of cells will spread, ultimately replacing the wild-type, at a rate dependant upon the growth advantage it has. This growth advantage is itself a variable dependant upon the selective pressure. Thus, for a given mutation with a frequency of 1/N, where N is the number of cells in the population, the ability of the observer to detect an overall population of cells with the altered growth rate may appear to be stepwise although it is likely to be a reflection of changes in the frequency of the mutant in the population, since it takes as long to change from a frequency of 1/1000 to 1/100 as it takes to change from 1/100 to 1/10, with the former change likely undetectable, whereas the latter would be visible by the observer. This issue would be further complicated by instances in which multiple variants with ranges of growth advantages might arise, resulting in mixed populations of variants at discrete stages, although presumably if the selection occurs for long enough one variant will take over.

It is likely that more than one evolutionary pathway to thermotolerance exists and the Evolugator™ could be used to probe this interesting question. Essentially, the Evolugator™ selects for variants with positive growth rates over those with zero or negative growth rates. During our adaptation experiment it was noted that it takes longer for favorable variants to take over during certain cycles, appearing to indicate that the evolution is occurring in discrete steps, although this may be inaccurate. For example, we observed that for most incremental increases in temperature, the selection for faster growing variants was rapid and took roughly the same amount of time. However, at certain temperatures (32°C, 36.5°C and 37.5°C), it took longer for favorable variants to take over, hence these temperatures were considered as thermal barriers, perhaps requiring multiple or complex mutations to arise in the population. It is possible that a different evolutionary pathway might encounter different thermal barriers. More experiments are needed to determine if these temperatures represent concrete barriers or if they are an artifact of the peculiar evolutionary pathway taken in our experiment. It will also be important to compare the genome sequence of the thermotolerant variants with that of wild type to determine the genetic changes involved in the acquisition of thermotolerance.

## Conclusion

We report the successful directed adaption of a eukaryotic organism, namely the filamentous fungus, *M. anisopliae*, for increased thermotolerance using a novel continuous culture machine whose operation is fundamentally different from chemostats and other current methods. The selection regime had no *a priori *suppositions regarding the mutation(s) required and it remains to be seen how many different mutational pathways can lead to thermotolerance. Experiments isolating additional thermotolerant mutants should be able to shed light on this issue. Selected mutants displayed complex phenotypes with respect to non-selected attributes such as conidiation and virulence, although both parameters could be retained or recovered while maintaining the thermotolerant phenotype.

The immediate goal of future research will be to generate "designer" strains of entomopathogenic fungi that are more efficient biocontrol agents than currently used wild type strains for specific insect infestations and defined environmental conditions. Such strains would have significant advantages over other types of insecticide currently in use. First, because these strains are more specific for insect species and environmental conditions, less agent would be required to achieve the same result. Second, since they are biological control agents, they would lack the environmental impact of chemical pesticides. Third, these strains would be generated by the same mechanism that drives natural selection and, consequently, they will not carry the stigma of genetically-engineered products.

In theory, this technology can be applied for the adaptation of any cultivatable eukaryotic organism to specific selection and growth conditions. For example, the thermostability of eukaryotic microorganisms remains an important engineering constraint in a variety of biocatalytic applications, such as the conversion of lignocellulosic biomass to desirable end-products like biofuels. Accordingly, long-term research goals involve expanding the use of this technology to other important biotechnological problems.

## Methods

### Isolation of thermotolerant *M. anisopliae *and determination of growth kinetics

The details of how the Evolugator™ achieves continuous culture and the inherent advantages of this technology over other methods of continuous culture are extensively described elsewhere [6]. However, since the technology is so new, it is important to summarize how it works. Briefly, directed selection occurs inside a growth chamber made of 100% silicone tubing (12.7 mm external diameter and 9.5 mm internal diameter, Saint Gobain, France) that is flexible, transparent and gas-permeable. The tubing is filled with growth medium and sterilized prior to mounting into the Evolugator™, where it is subdivided using "gates", which are clamps that prevent the flow of medium and cultured organisms from one subdivision to the next. Between the central gates is the "growth chamber", which has a volume of ~10.8 mL. Oxygenation of the growth chamber is augmented beyond the permeability of the tubing by maintaining a 1.8 mL (± 5%) bubble of filtered air in the growth chamber. Cultures are inoculated into the growth chamber through the tubing using sterilized syringes. The growth medium and the inner surface of the tubing are static with respect to each other, and both are regularly and simultaneously replaced by peristaltic movement of the tubing through the gates. A fresh air bubble is delivered with each dilution cycle by movement of air in predetermined volumes through the unused portion of media upstream of the growth chamber.

In summary, the gates are periodically released allowing unused medium to mix with saturated culture. The tubing is then moved and the gates reclosed – essentially, the majority of the medium and growth chamber are entirely replaced during every dilution cycle. In the "new growth chamber", culture is diluted with unused medium. The "old' growth chamber is now what is called the "sampling chamber" from which samples can be extracted by syringe without fear of contaminating the "new growth chamber".

Dilutions were conducted automatically and controlled through specifically designed software. Dilution can be initiated at a certain cycle duration (chemostat mode), when the culture attains a certain OD (turbidostat mode) or a combination of both. Two turbidimeters (λ = 680 nm, power = 0.7 V) (EFS, Montagny, France) measure the optical density and are zeroed with unused growth medium prior to each experiment.

Since filamentous fungi adhere to solid surfaces, they grow along the inner surface of the "growth chamber". Since the cells from the previous cycle adhere closest to the gate separating the "sampling chamber" and the "new growth chamber", dividing cells will grow along the fresh chamber surface towards the gate separating the "new growth chamber" from unspent medium. Consequently, the cells that reach this gate by growing along the surface are the most recent (and presumably most fit) additions to the population and will be retained in the active culture when the tubing moves again to achieve the next dilution.

For directed evolution of *M. anisopliae*, the tubing was filled with Sabouraud dextrose (SAB) media and autoclaved prior to use. 2 mL of a growing culture of *M. anisopliae *2575 grown in SAB was injected into the first section of the growth chamber and dilution cycles were initiated as described. Temperature was monitored using a PT100 probe (IEC/Din Class A) and regulated via a Proportional Integral & Derivative controller (West P6100). Growth kinetics were determined using a Bioscreen C plate reader system (Growth Curves USA, Piscataway, NJ) in multiple volumes of 250–300 μL. Aliquots of growing cultures were mounted on slides and examined using a PASCAL LSM5 confocal microscope fitted with Nomarski differential interference contrast (DIC) optics.

### Identification of recovered adapted fungal strains

Single isolated fungal colonies (corresponding to EVG016, EVG017, and EVG017g) were re-streaked onto fresh Potato dextrose agar plates and used for identification purposes. Fungal identity was confirmed by PCR amplification and sequencing of a portion of the 5.8 S rRNA with its flanking internal transcribed spacer sequences (ITS) and the *M. anisopliae *specific protease Pr1 as described [35,36]. Primer pairs used were: (1) ITS5; 5'-gcaagtaaaagtcgtaacaagg, and ITS4; 5'-tcctccgcttattgatatgc-3' and (2) Pr1f, 5'-gccgacttcgtttacgagcac, and Pr1r, 5'-ggaggcctcaataccagtgtc. Genomic DNA was isolated using the Qiagen DNeasy Plant mini-extraction kit according to the manufacturer's protocols (Qiagen Inc., Valencia, CA). PCR reactions were performed using ExTaq DNA polymerase (Takara Corp., obtained from Fisher Sci, Pittsburgh, PA). PCR products were cloned into the pCR 2.1-TOPO vector (Invitrogen, Carlsbad, CA) according to the manufacturer's protocols. Plasmid inserts were sequenced at the University of Florida, ICBR, Sequencing Facility.

### Evaluation of conidial production and production of conidia for bioassays

Conidia of *M. anisopliae *Strain ARSEF2575 and the two temperature tolerant clones, EVG016 and EVG017, were produced in a biphasic system by ARS at Sidney MT following the methods of Bradley et al 1992[37]. In brief, conidia from agar media were used to inoculate flasks of liquid medium (40 g L^-1 ^glucose, 20 g L^-1 ^yeast extract, 15 ml L^-1 ^corn steep liquor). The liquid cultures were incubated for 3–4 d at 25–26°C and 150 rpm and then used to inoculate flaked barley (Minnesota Grain, Eagan MN) that had been premoistened with reverse osmosis water (3:5 v:w), and autoclaved (103 KPa for 30 min/kg) in vented, plastic mushroom spawn bags (SacO2, Microsac, Belgium). The liquid cultures were mixed with the substrate within the bags under aseptic conditions, at a ratio of 3:10 (v/w) and the open ends of the bags were heat-sealed. The solid substrate fermentation phase proceeded at 26–27°C in constant darkness. Cultures were observed daily and crumbled by hand within spawn bags as needed to prevent binding of the substrate and provide aeration throughout the culture substrate. After 8–10 days the whole cultures were then transferred to kraft paper bags in which they were dried for 10 days at 23–25°C, to a final moisture of < 0.4 Water Activity Units (< 6% gravimetric moisture). Conidia were harvested by mechanical sieving through 20-and 100-mesh sieves under standardized conditions in an ultrasonic sieve shaker (AS200, Retsch Corp., Newton PA) with the conidial fractions < 0.150 mm (100 U.S. Mesh) retained for yield determination and use in bioassays. After conidia were harvested, the mass of harvested conidia and conidial counts were used to calculate yield per kg of dry barley. Three replicate batches of 500 g dry barley were used for each strain as described above. For conidial counts two replicate samples of 0.1 g of harvested conidia were suspended in 0.1% Silwet L77™ (Loveland Chemicals), serially diluted with water as appropriate and counted using an Improved Neubauer hemocytometer under 400× phase contrast microscopy. All conidial preparations were stored at 3°C until use.

### Insect Bioassays

Prior to use in bioassays, conidial viabilities were determined by plating dilute aqueous suspensions of each technical powder onto potato dextrose agar, incubating at 27–28°C for 16–19 hr, and then examining the conidia with 400× phase contrast microscopy. A preliminary step to determine fungal viability was performed, in which a small quantity of dry conidia was exposed to 100% relative humidity for 1–2 hr before suspension and plating. A minimum of 400 conidia were examined for germination; a conidium was considered viable (germinated) if it had produced a visible germination peg during the specified incubation time. Viabilities of the two *M. anisopliae *technical powders were 90 and 92% for 2575 and EVG016, respectively. Because the conidial production of the original EVG017 was insufficient to provide enough conidia for bioassay, the clone was passed through adult *Melanoplus sanguinipes*, reisolated from a single colony and grown for two cycles on agar media. The passaged fungus, EVG017g, regained ability to sporulate on Sabouraud dextrose agar supplemented with 0.1% yeast extract (SDAY) and Potato dextrose agar (PDA), as well as in solid substrate fermentation and was used to evaluate conidial production and to produce conidia for bioassays as described above. Conidial viability of the conidia powder used for bioassays was 88%.

For bioassays, the dry conidial powders were first formulated in culinary canola oil with the final titers determined by hemocytometer counts of serial dilutions made with kerosene. To determine the relative infectivity (median infectious dose) and virulence (median and average lethal times of one selected dose), a series of conidial suspensions in canola were prepared with the actual spore concentrations determined by hemocytometer count and adjusted for conidial viability. One week old, adult *M. sanguinipes *from a nondiapausing laboratory colony were used in all bioassays. A topical, 5-dose bioassay with adult *Melanoplus sanguinipes *was conducted with doses bracketing the estimated LD_50 _for each strain, based on a preliminary bioassay. In the bioassay a one μl droplet of spores in oil was applied to the front left coxa of each insect, with 20–25 insects per dose. Dosed insects and controls incubated at 28°C in cylindrical cellulose acetate cages (50 cm × 10 cm) with mesh covered openings. Bioassays were replicated twice in their entirety, with a total of 120–150 insect per bioassay. Day 7 mortalities were used to calculate LD_50 _and associated statistics by probit analysis with Polo-Plus™ (LeOra Software). Median and average survival times were calculated using Kaplan Meier survivorship statistics [34] with KMSsurv.exe [38]. The replicate tests for each fungus were first compared, and, being not significantly different were combined for further analysis.

To determine the heat tolerance of the parent and both mutant strains *in vivo*, adult *M. sanguinipes *were dosed topically at the ~LD_95 _for each clone or parent and incubated at 36 ± 0.5°C or 28 ± 0.5°C. Daily mortality was recorded for 14 days. Temperatures were monitored continuously by means of a Hobo^® ^temperature logger placed in an empty grasshopper container, which was positioned amidst the grasshopper containers. All assays were replicated twice in their entirety. Median and average lethal times were calculated as described above. The ST_50 _for each strain was calculated as described above for the 28°C treatments; this parameter could not be calculated for the higher temperature because of the low mortalities. Insect body temperatures were monitored with a thermocouple inserted into a thermal surrogate placed within an empty cage along with the cages with grasshoppers, a technique that has been shown to accurately reflect grasshopper body temperatures during both normal and thermoregulatory behavior [39,40].

Conidial germination and radial growth studies of the three fungi were conducted in parallel with the insect bioassays to better understand the assay mortalities. The reisolated EVG017g was evaluated along with the original ARSEF2575 and EVG016 fungi. Conidial germination tests were conducted as the viability determinations described earlier but parallel plates were incubated at 36.5°C as well as 28°C and conidia examined at 18, 24, 48, 72 and 96 hr. After the last observation the Petri plates of fungi incubated at 36.5°C were transferred to 28°C and observed daily for another 2–3 days.

Radial growth tests were carried out in several ways. Replicate 60 mm Petri plates of SDAY agar were point-inoculated with the fungi, and initially incubated at 36.5°C. for 4 days, then half of the samples were transferred to 28°C for further observation. Other plates were incubated at 28°C until the colonies were 3–4 mm in diameter at which time one half were transferred to 36.5°C and subsequent radial growth monitored for up to 18 days. In all cases, colony radii were measured daily across two perpendicular axes with a digital Vernier caliper. There were three replicate plates for each treatment.

## Authors' contributions

EC and NK conceived the experiments, analyzed the data and wrote the paper. EC conducted the evolution experiment. SJ conducted the conidial production studies, fungus passage and reisolation steps, insect bioassays, and *in vitro *radial growth experiments, BL, and TL performed assays, assisted in the evolution experiment, assisted in analyzing the data and writing the paper.

## References

[B1] Arora DK, ed (2003). Handbook of Fungal Biotechnology.

[B2] Pointing SB, Hyde KD, eds (2001). Bio-exploitation of filamentous fungi.

[B3] Streit WR, Daniel R, Jaeger KE (2004). Prospecting for biocatalysts and drugs in the genomes of non-cultured microorganisms. Current Opinion in Biotechnology.

[B4] Riehle MM, Bennett AF, Lenski RE, Long AD (2003). Evolutionary changes in heat-inducible gene expression in lines of Escherichia coli adapted to high temperature. Physiological Genomics.

[B5] Witten JT, Chen CT, Cohen BA (2007). Complex genetic changes in strains of Saccharomyces cerevisiae derived by selection in the laboratory. Genetics.

[B6] de Crecy E, Metzgar D, Allen C, Penicaud M, Lyons B, Hansen CJ, de Crecy-Lagard V (2007). Development of a novel continuous culture device for experimental evolution of bacterial populations. Applied Microbiology and Biotechnology.

[B7] Kohn LM, Schaffer MR, Anderson JB, Grunwald NJ (2008). Marker stability throughout 400 days of in vitro hyphal growth in the filamentous ascomycete, Sclerotinia sclerotiorum. Fungal Genetics and Biology.

[B8] Larsen B, Poulsen BR, Eriksen NT, Iversen JJL (2004). Homogeneous batch cultures of Aspergillus oryzae by elimination of wall growth in the Variomixing bioreactor. Applied Microbiology and Biotechnology.

[B9] de Faria MR, Wraight SP (2007). Mycoinsecticides and mycoacaricides: A comprehensive list with worldwide coverage and international classification of formulation types. Biological Control.

[B10] Biopesticide Active Ingredient Fact Sheets. http://www.epa.gov/pesticides/biopesticides/ingredients/.

[B11] Goettel MS, Eilenberg J, Glare TR, Gilbert LI, Iatrou K, Gill S (2005). Entomopathogenic Fungi and their role in regulation of insect populations. Comprehensive Molecular Insect Science.

[B12] Kirkland BH, Westwood GS, Keyhani NO (2004). Pathogenicity of entomopathogenic fungi *Beauveria bassiana *and *Metarhizium anisopliae *to Ixodidae tick species *Dermacentor variabilis, Rhipicephalus sanguineus*, and *Ixodes scapularis*. J Med Entomol.

[B13] Roberts DW, St Leger R (2004). Metarhizium spp., Cosmopolitan Insect-Pathogrnic Fungi: Mycological Aspects. Advances in Applied Microbiology.

[B14] Kirkland BH, Cho EM, Keyhani NO (2004). Differential susceptibility of *Amblyomma maculatum *and *Amblyomma americanum *(Acari: Ixodidea) to the entomopathogenic fungi *Beauveria bassiana *and *Metarhizium anisopliae*. Biological Control.

[B15] Barrientos-Lozano L, Hernandez-Velazquez VM, Milner RJ (2002). Advances in biological control of locusts and grasshoppers in Mexico. Journal of Orthoptera Research.

[B16] Blanford S, Chan BH, Jenkins N, Sim D, Turner RJ, Read AF, Thomas MB (2005). Fungal pathogen reduces potential for malaria transmission. Science.

[B17] Milner RJ, Hunter DM (2001). Recent developments in the use of fungi as biopesticides against locusts and grasshoppers in Australia. Journal of Orthoptera Research.

[B18] Inglis GD, Goettel MS, Butt TM, Strasser H, Lacey LA, Kaya HK (2001). Use of hyphomycetous fungi for managing insect pests. Field manual of techniques in invertebrate pathology.

[B19] Scholte EJ, Knols BG, Takken FL (2006). Infection of the malaria mosquito *Anopheles gambiae *with the entomopathogenic fungus *Metarhizium anisopliae *reduces blood feeding and fecundity. Journal of Invertebrate Pathology.

[B20] Rangel DE, Alston DG, Roberts DW (2008). Effects of physical and nutritional stress conditions during mycelial growth on conidial germination speed, adhesion to host cuticle, and virulence of Metarhizium anisopliae, an entomopathogenic fungus. Mycological Research.

[B21] Rangel DE, Braga GU, Anderson AJ, Roberts DW (2005). Variability in conidial thermotolerance of Metarhizium anisopliae, isolates from different geographic origins. Journal of Invertebrate Pathology.

[B22] Roy HE, Steinkraus DC, Eilenberg J, Hajek AE, Pell JK (2006). Bizarre interactions and endgames: Entomopathogenic fungi and their arthropod hosts. Annual Review of Entomology.

[B23] Chown SL, Addo-Bediako A, Gaston KJ (2002). Physiological variation in insects: large-scale patterns and their implications. Comparative Biochemistry and Physiology B-Biochemistry & Molecular Biology.

[B24] Gardner SN, Thomas MB (2002). Costs and benefits of fighting infection in locusts. Evolutionary Ecology Research.

[B25] Heinrich B (1995). Insect Thermoregulation. Endeavour.

[B26] Fargues J, Goettel MS, Smits N, Ouedraogo A, Rougier M (1997). Effect of temperature on vegetative growth of Beauveria bassiana isolates from different origins. Mycologia.

[B27] Fargues J, Goettel MS, Smits N, Ouedraogo A, Vidal C, Lacey LA, Lomer CJ, Rougier M (1996). Variability in susceptibility to simulated sunlight of conidia among isolates of entomopathogenic Hyphomycetes. Mycopathologia.

[B28] Ouedraogo A, Fargues J, Goettel MS, Lomer CJ (1997). Effect of temperature on vegetative growth among isolates of Metarhizium anisopliae and M-flavoviride. Mycopathologia.

[B29] Wang C, St. Leger R (2007). A scorpion neurotoxin increases the potency of a fungal insecticide. Nature Biotechnology.

[B30] Kirkland BH, Eisa A, Keyhani NO (2005). Oxalic acid as a fungal acaracidal virulence factor. Journal of Medical Entomology.

[B31] Kurtti TJ, Keyhani NO (2008). Intracellular infection of tick cell lines by the entomopathogenic fungus Metarhizium anisopliae. Microbiology.

[B32] St. Leger R, Joshi L, Bidochka MJ, Roberts DW (1996). Construction of an improved mycoinsecticide overexpressing a toxic protease. Proceedings of the National Academy of Science.

[B33] Wraight SP, Jackson MA, De Kock SL, Butt TM, Jackson CW, Magan N (2001). Production, stabilization, and formulation of fungal biocontrol agents. Fungi as Biocontrol Agents: Progress, Problems, and Potential.

[B34] Klein JP, Moeschberger (1997). Survival Analysis: Techniques for Censored and Truncated Data.

[B35] White TJ, Bruns T, Lee S, Taylor J, Innis M, White M, Sninsky JJ (1990). Amplification and direct sequencing of fungal ribosomal RNA genes for phylogentics. PCR Protocols: A Guide to Methods and Applications.

[B36] Stleger RJ, Frank DC, Roberts DW, Staples RC (1992). Molecular-Cloning and Regulatory Analysis of the Cuticle-Degrading-Protease Structural Gene from the Entomopathogenic Fungus Metarhizium-Anisopliae. European Journal of Biochemistry.

[B37] Bradley CA, Black WE, Kearns R, Wood P, Leatham GE (1992). Role of production technology in mycoinsecticide development. Frontiers in Industrial Microbiology.

[B38] Campos N, Franco EL (1988). Microcomputer assisted univariate survival data analysis using Kaplan-Meier life table estimators. Computer Methods and Programs in Biomedicine.

[B39] Lactin DJ, Johnson DL (1996). Behavioural optimization of body temperature by nymphal grasshoppers (Melanoplus sanguinipes, Orthoptera: Acrididae) in temperature gradients established using incandescent bulbs. Journal of Thermal Biology.

[B40] Lactin DJ, Johnson DL (1996). Effects of insolation and body orientation on internal thoracic temperature of nymphal Melanoplus packardii (Orthoptera: Acrididae). Environmental Entomology.

